# Comparison of Cost and Potency of Human Mesenchymal Stromal Cell Conditioned Medium Derived from 2- and 3-Dimensional Cultures

**DOI:** 10.3390/bioengineering10080930

**Published:** 2023-08-04

**Authors:** Marialaura Madrigal, Patricia L. Fernández, Ricardo Lleonart, Lizmar Carreño, Kaiser Alejandro Villalobos Gorday, Ellís Rodríguez, Kathya de Cupeiro, Carlos M. Restrepo, K. S. Jagannatha Rao, Neil H. Riordan

**Affiliations:** 1 MediStem Panama Inc., Panama City 7144, Panama; 2 Department of Biotechnology, Acharya Nagarjuna University, Guntur 522510, India; 3 Centro de Biología Celular y Molecular de Enfermedades, Instituto de Investigaciones Científicas y Servicios de Alta Tecnología (INDICASAT-AIP), Panama City 7144, Panama; 4 Department of Biotechnology, Konenru Lakshmaiah Education Foundation (KLEF) deemed to be University, Vaddeswaram 522302, India

**Keywords:** UC-MSC, trophic factors, conditioned medium, tridimensional culture, bioreactor, mesenchymal, MSC, potency test, anti-inflammatory effect, TRegs, DMARDs, NSAIDs, cost effective, COVID-19, auto-immune conditions, inflammation

## Abstract

Mesenchymal stromal cell (MSC)-derived products, such as trophic factors (MTFs), have anti-inflammatory properties that make them attractive for cell-free treatment. Three-dimensional (3D) culture can enhance these properties, and large-scale expansion using a bioreactor can reduce manufacturing costs. Three lots of MTFs were obtained from umbilical cord MSCs produced by either monolayer culture (Monol MTF) or using a 3D microcarrier in a spinner flask dynamic system (Bioreactor MTF). The resulting MTFs were tested and compared using anti-inflammatory potency assays in two different systems: (1) a phytohemagglutinin-activated peripheral blood mononuclear cell (PBMNC) system and (2) a lipopolysaccharide (LPS)-activated macrophage system. Cytokine expression by macrophages was measured via RT-PCR. The production costs of hypothetical units of anti-inflammatory effects were calculated using the percentage of TNF-α inhibition by MTF exposure. Bioreactor MTFs had a higher inhibitory effect on TNF (*p* < 0.01) than monolayer MTFs (*p* < 0.05). The anti-inflammatory effect of Bioreactor MTFs on IL-1β, TNF-α, IL-8, IL-6, and MIP-1 was significantly higher than that of monolayer MTFs. The production cost of 1% inhibition of TNF-α was 11–40% higher using monolayer culture compared to bioreactor-derived MTFs. A 3D dynamic culture was, therefore, able to produce high-quality MTFs, with robust anti-inflammatory properties, more efficiently than monolayer static systems.

## 1. Introduction

Inflammation plays a critical role in the modulation and healing of acute injuries, but may become chronic due to an aberrant response long after the initial injury or insult [1,2]. Inflammation-related diseases, a leading cause of disability and mortality worldwide, can result in decreased quality of life [3,4,5]. Medications currently available to treat these diseases may have adverse effects and be cost-prohibitive [6,7]. In the past decade, mesenchymal stromal cell (MSC) therapy has been studied in several clinical trials for autoimmune conditions. Due to the immunomodulatory characteristics and anti-inflammatory capacity of MSCs [8], MSC therapy has emerged as a promising approach for autoimmune disorders [9,10,11] and chronic inflammation [12,13]. MSCs, initially isolated from bone marrow in 1987 [14], can also be obtained from various sources, such as adipose tissue, perinatal products (amniotic fluid, placenta, umbilical cord), dental pulp, and stromal vascular fraction, among others [15,16]. MSCs are notably easier to obtain from the Wharton’s jelly of human umbilical cords (UC-MSCs), which are typically discarded after birth, than those derived from other tissues. UC-MSCs allow for larger scale expansion and potentially result in better clinical outcomes than MSCs from other sources [17].

While the safety of MSC therapy has been demonstrated in some trials, there is an expanding industry [18], and also a growing interest, in developing cell-free products to eliminate the potential risk of immunogenicity, as well as certain side effects and risks, such as emboli formation [19,20]. The therapeutic effects of MSCs can be given by cell–cell contact, but are also mediated by their secretions of biomolecules, such as growth factors (HGF-1, TGF-β, VEFG), tumor necrosis factor-stimulated gene-6 (TSG-6), PGE2, interleukins (IL-1Ra, IL-6, IL-10, IL-13) [21], galectins, and extracellular vesicles [22], among others. MSC-derived exosomes, for example, have well-documented anti-inflammatory and immunomodulatory functions [23]. Moreover, using MSC-derived trophic factors (MTFs) in conditioned media has yielded results comparable to those using MSCs [24]. However, measuring only one molecule or extracellular vesicle may not be sufficient to determine dosage or to select lots for a therapeutic product [23,25]. Obtaining a consistent therapeutic benefit from an MSC product requires identifying specific markers for the desired therapeutic effect [26], assessing the product’s potency, and, ideally, minimizing production costs.

Different culture conditions induce metabolic changes. The 3D culture is known to increase mitochondrial activity and stemness when compared to 2D culture conditions [27]. The 3D culture may also influence the effects of MSCs as anti-inflammatory agents [22]. Specifically, 3D culture has been demonstrated to improve the secretion of the anti-inflammatory molecules prostaglandin E2 (PGE2) and tumor necrosis factor (TNF)-stimulated gene 6 (TSG-6) [28,29]. A bioreactor culture allows pH and glucose to be monitored, while nutrients and oxygen are equally distributed to all cells. These conditions reduce labor hours and allow for more space-effective production. The use of closed-system bioreactors translates into production cleanrooms with less stringent requirements for cell therapy medicinal products (CTMPs) with good manufacturing practices (GMPs), thereby reducing production costs compared to expansion using flasks in an open system, which increases the cost of aseptic environment conditions and carries higher risks of contamination [30,31]. It is, thus, desirable to find a compromise among quality, efficacy, and cost in choosing culture methods.

Finally, a potency assay is an invaluable tool for monitoring inter-batch consistency. Inflammatory models using peripheral blood mononuclear cells, lymphocytes, and macrophages have been used to test MSC-secreted factors that appropriately mimic in vivo conditions [32,33]. UC-MSCs increase the frequency of CD4+, CD25high, and CD45RA+ Tregs in phytohemagglutinin (PHA)-activated peripheral blood mononuclear cells (PBMNCs) [34]. Likewise, MSC-derived exosomes on PHA-stimulated PBMNCs modulate the gene expression of B Lymphocytes and affect T-cell activation [35]. MSC potency may also be evaluated in macrophages primed with LPS to test the immune-modulatory effect of exosomes [33,36]. In the present investigation, both approaches were used for potency tests of the MTFs obtained from UC-MSC conditioned medium.

Before translating to large-scale expansion bioreactors, it is necessary to perform a small-scale evaluation of the conditions of culture, including agitation, culture media, and, in the case of MSCs, which are anchorage-dependent cells, the microcarriers. In this work, we choose a disposable spinner flask and gelatin microcarriers (dynamic 3D cultivation).

We aimed to evaluate the reliability of dynamic 3D microcarriers culture in producing MTFs and to compare this method with monolayer culture (static 2D cultivation). Additionally, we aimed to determine the anti-inflammatory potency and the immunomodulatory effects of MTFs derived from 3D culture. The goal of the research was to contribute to quality control and release criteria standardization based on in vitro potency tests [37], and to investigate the efficiency of 3D vs. 2D UC-MSC conditioned medium in producing inflammatory properties.

## 2. Materials and Methods

### 2.1. Cell Culture and MTFs Production

The UC-MSCs used in this study were obtained from MediStem Panama Inc. (Panama City, Panama), a biotechnology facility. Vials from their R&D department cell master bank were obtained in 2014 under informed consent, and the investigators did not have access to identifying information of the biospecimens used for this study. The process of reviewing informed consent and anonymization of the biospecimens was overseen by IRCM IRB (IRCM-2023-372). These cells were previously frozen in passage 2; characterized (positive for CD90, CD73, and CD105 and negative for CD45 and CD34); and passed the differentiation potential tests (chondrogenic, adipogenic, and osteogenic), according to the minimal criteria established by the International Society for Cell & Gene Therapy (ISCT) [38] (Supplementary Figures S1–S3).

**Figure 1:**
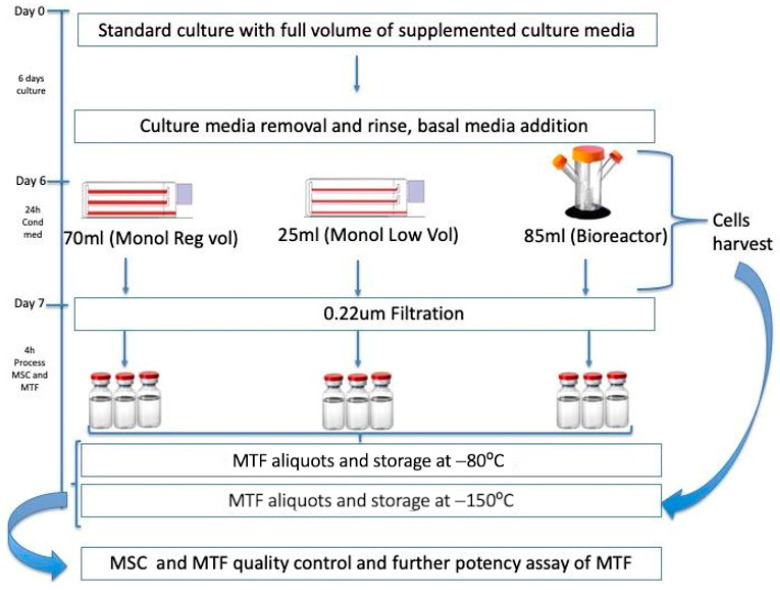
Workflow schematics. Experimental procedure for MSC culture and MTFs collection from spinner flask (bioreactor) or monolayer cultures using regular- and low-volume media. The process begins with cells cultured over six days either on monolayer (tissue culture flasks) or on gelatin microcarriers in the 125 mL spinner flask bioreactor. Then, media were changed in Triple Flasks either with 70 mL per flask or with 25 mL per flask to obtain MTFs from Monol Reg Vol or Monol Low Vol, respectively, using serum-free media from day 6 to day 7 (24 h incubation). To collect Bioreactor MTFs, 85 mL of the same serum-free media was added to the spinner flask; after 24 h, the conditioned media were removed, filtered, aliquoted, and frozen. Cells were harvested, counted, and cryopreserved in aliquots for further testing. The same procedure was completed for monolayer MTFs.

Three lots were used to perform the experiments under two conditions (Figure 1): (1) monolayer culture in tissue culture-treated flasks with two different volumes of conditioned medium and (2) 3D culture in a 125mL disposable spinner flask (Corning, Darmstadt, Germany) with gelatin Cultispher S (Sigma, Darmstadt, Germany) microcarriers in a bioreactor.

The monolayer culture was inoculated with 5–5.5 × 10^4^ cells/cm^2^ (1.33 × 10^6^ cells/flask), using passage 2 UC-MSCs in T175 flasks (BD Falcon, Nunc, Roskilde, Denmark). On day 3, the culture was divided into thirds using TrypLE™ Express 1X, and cells were re-inoculated into the TripleFlask^TM^ (NUNC). The division was performed again on day 5, resulting in a total of four TripleFlasks^TM^. Culture medium was removed on day 6 and replaced by serum-free RPMI medium with 2 mM GlutaMAX, using 70 mL of medium (Monol Reg Vol) in one flask and 25 mL (Monol Low Vol) in the other two.

For the 3D microcarriers dynamic cultures, 0.35 g of Cultispher-S microcarriers were prepared, following the manufacturer’s instructions. A total of 1.33 × 10^6^ passage 2 UC-MSCs were inoculated under intermittent agitation for 8 h (3 min at 80 rpm; 27 min at rest), using MEM alpha supplemented with 4 mM GlutaMAX and 2% FBS. Following the 8 h period, FBS was added to reach a final concentration of 10% in a total volume of 100 mL. During the culture processing, agitation was kept at 60 rpm, and glucose was monitored twice a day using CellGlucose^TM^ strips (Cesco Bioengineering, Trevose, PA, USA). Glucose levels were maintained above 60 mg/dL by partial changing of the medium (25–50%). On day 5, the final volume was adjusted to 125 mL. Bioreactor cultures were visually inspected; taking a small aliquot, microcarriers were stained with acridine orange to confirm growth and confirm absence of apoptosis (Supplementary Figure S4).

On day 6, all media were removed; microcarriers (with attached cells) were washed with PBS twice, using cell strainers of 70 μm mesh to retain the microcarriers; then, those microcarriers were returned to the spinner flask and resuspended in 85 mL of serum-free media, using RPMI 1640 (Lonza, Bend, OR, USA) without phenol red, supplemented with 2 mM GlutaMAX. The cells in the microcarriers were incubated under normoxic (5% CO_2_, 37 °C, 100% humidity) conditions at an agitation speed of 60 rpm.

On day 7, after 24 h of additional incubation, MTFs were collected from the conditioned medium of all three culture setups by filtering them through a 0.2 μm PES low-protein binding filter (Nalgene); the MTFs were then aliquoted, protected from light, and kept at −80 °C for further analysis. Monolayer cultured UCMSCs were washed with PBS; then, flasks were tripsinized using TrypLE Express 1X and incubating for 6 min at 37 °C. The bioreactor microcarriers were dissolved using TrypLE ™ Select 2X, using incubation at 37 °C and 120 rpm speed agitation for 15–20 min. The cells from each system were collected in 50 mL conical tubes, then washed twice with PBS and counted, and viability was determined using Guava^®^ ViaCount^TM^ Reagent (Luminex Corp., Austin, TX, USA) in a flow microcytometer (Guava^®^ easyCyte^TM^ 8HT), using the validated method against Trypan blue. Then, the cells were frozen using a 6% pentastarch in saline (NaCl 0.9%) solution with 10% dimethyl sulfoxide (DMSO).

Cells grown in the bioreactor and in monolayer were compared in terms of doublings per day, which was calculated with the following Equation (1):

X = ((log(n) − log(n0))/log(2))/days(1)

where n = number of cells obtained, and n0 = number of cells inoculated

The concentration of total protein in each MTF was measured using fluorometry with the Qubit total protein detection kit, corrected for use with the MTF matrix, and properly validated (Qubit, Thermo, Waltham, MA, USA). PGE2 and IL-6 were measured quantitatively with ELISA (PGE2 (Cayman Chemical, Ann Arbor, MI, USA); IL-6 (Thermo)). The cell number per mL was also determined for each MTF based on the harvested MSCs count and the volume obtained. To compare magnitudes among the three setups without depending on the number of cells needed to produce a milliliter of MTFs, the values were reported as production per million cells.

### 2.2. In Vitro Potency Assays for MTFs Using PHA on PBMNCs and Macrophages with LPS

MTFs from the monolayer and bioreactor procedures were subjected to potency tests in three setups: (a) PBMNC activated with phytohemagglutinin (PHA); (b) THP-1-derived macrophages activated with lipopolysaccharides (LPS) at the same time as MTFs (Treatment 1); or (c) THP-1-derived macrophages activated with lipopolysaccharides (LPS) added before MTFs (Treatment 2) (See Appendix A for additional details).

For the PHA inflammation assay, PBMNCs were incubated with 5 μg/mL PHA, using 50% MTFs for 48 h; supernatants were collected for TNF-α ELISA tests. The other in vitro model was THP-1-derived macrophages incubated with MTFs for 5.5 h in two treatment setups. For Treatment 1 (T1), MTF was added with LPS during the 5.5 h. For Treatment 2 (T2), cells were pre-treated with LPS for 90 min; then, MTF was added, and the cells were incubated for 4 more hours. LPS was used as a control of activation, and dexamethasone was used as a positive control of inhibition. After these treatments, supernatants were collected for TNF-α ELISA assays, and the macrophages were characterized in a flow cytometer. Cell lysates were used for further RNA extraction. RT-PCR tests were performed to quantify inflammatory cytokine expression.

RT-PCR was performed using SYBR Green. β-Actin expression served as the reference gene for the relative quantification. Five inflammatory mediators (IL-6, IL-1β, TNF-α, IL-8, and MIP-1) were evaluated. Appendix A provides more details of this methods section.

### 2.3. Statistical Analysis

Bioreactor and monolayer data set results were analyzed using one-way ANOVA and a Tukey post hoc test with Graph Pad (PRISM 9), with *p* < 0.05 considered statistically significant. The mRNA folding induction was calculated based on the Ct values obtained from the Applied Biosystems 7500 analyzer tool for comparative Ct. To obtain the relative expression of the cytokines, a 2-ΔΔCt (delta Ct) analysis was used, with β-actin gene expression serving as the reference. The mRNA folding induction data were grouped according to whether they were treated with LPS (+LPS) or not (−LPS), for each gene separately. A two-way ANOVA was run, as well as Tukey multiple comparison tests of the means of the different conditions (−LPS or +LPS), with an alpha of 0.05. Then, a normalization was performed, using percentages of mRNA folding induction, assuming that control +LPS (without MTF) corresponded to 100% expression of the molecule for each assay. Since −LPS samples do not overexpress inflammatory cytokines, those were obviated for this second set of figures. 

Results for 5 different genes are presented as means with standard error of the mean (SEM) for the THP-1 macrophages assay. Outliers were identified using ROUT −1% analysis (7 numbers were removed from a data set of 590 values). A one-way ANOVA was performed with a Tukey post hoc multiple comparison (confidence interval CI = 95%) to infer statistically significant differences between the anti-inflammatory effects of different MTFs or dexamethasone using Graph Pad version 9.0 (GraphPad Software, San Diego, CA, USA).

To obtain a quality control that is easy to implement and validate, an analysis with many variables is not desirable. Therefore, to study the behavior of the cytokines evaluated in each culture system and to establish a formula for a one-parameter potency test, the dimensionality of the data set was reduced by conducting a principal component analysis (PCA) using the R built-in function prcomp (R version 3.4.2). The PCA data were visualized in a factor map, using the R package factoextra version 1.0.5 [39] to identify correlated variables and their contribution to each of the two principal components. Comparing the correlation patterns of cytokines with their expected expression, the behavior was used as the criterion of inclusion or exclusion for each data set in different data sets derived from two treatments made on three batches of cells.

### 2.4. Comparison of the Cost of Each Anti-Inflammatory Unit Production

A spreadsheet was created to record the costs of every material, reagents, culture media, and hours required for both monolayer and bioreactor batch production, as well as for the quality control of the MTFs. Those costs were classified into the following categories: (a) Cells from the cell master bank (starting material); (b) Disposable plastic materials (disposable pipettes, tubes, flasks, and spinner flasks); (c) Reagents and microcarriers, including enzymes, buffers, metabolites monitoring, and microcarriers; (d) Culture media, which sums up the MEM alpha for the cell culture and the RPMI for MTF collection; (e) Quality control (QC)—all materials, reagents, and labor needed to finish the QC of each batch; (f) Labor hours of the cell culture and MTF process for each production system.

Total costs were then divided by the volume of MTFs produced to determine the cost per mL for each production type. The cost of one percent inhibition of TNF-α production from stimulated macrophages was then calculated using ELISA and RT-PCR tests, along with the cost per mL. This was done to compare the manufacturing systems and test the feasibility of a future bioreactor manufacturing system judged by the results on a spinner flask with 3D microcarriers culture at small scale.

## 3. Results

### 3.1. MTFs Produced in Different Culture Setups Are Compositionally Different

According to cell counts and viability determined at harvest, the total population doublings per day were between 0.70 and 0.90 for cell lots cultured in the 3D microcarrier spinner flask dynamic system. When converted to hours, this corresponds to one population doubling every 26.7 to 33.8 h. The average doubling time for each lot was 31.49 h (lot 1), 30.58 h (lot 2), and 31.21 h (lot 3). A 23-fold increase in cell count was observed following a 7-day culture. The viability percentage was above 85% for all batches of the cultured lots (Table 1). Doubling times and viability were very similar within the same lot and across the three lots tested. The lack of significant differences in these parameters validates the repeatability of the culture method established.

The number of cells required to yield one milliliter of MTFs was determined for each setup. We measured a mean of 1.7 × 10^5^ cells/mL of MTFs for the monolayer culture using regular-volume media (Monol Reg Vol), while the same measurement was 5.4 × 10^5^ cell/mL for the monolayer culture using low-volume media (Monol Low Vol) and 3.6 × 10^5^ cells/mL for the spinner flask with 3D microcarriers cultured cells.

**Figure 2:**
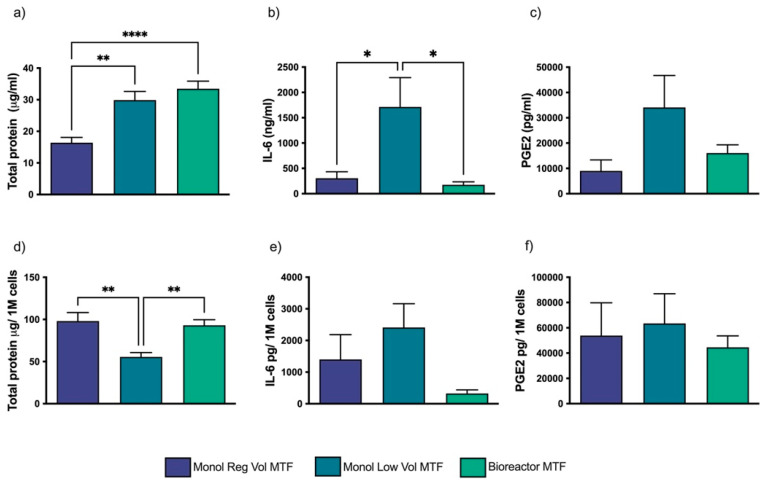
**MTF composition.** Concentrations (**a**–**c**) of total protein (µg/mL), IL-6 (ng/mL), and PGE2 (pg/mL), and production per million cells (**d**–**f**). * *p* < 0.05, ** *p* < 0.01, **** *p* < 0.001 with a one-way ANOVA test and multiple comparison of means using Tukey post hoc test. Error bars represent standard error of the mean (SEM).

The total protein concentration per mL was higher in the 3D microcarrier dynamic culture-produced MTFs (Bioreactor MTFs) (Figure 2a) and similar in magnitude per million cells when compared to that of the Monol Reg Vol cultures (Figure 2d). Although the total protein concentration per mL was similar between Bioreactor MTFs and Monol Low Vol MTFs, the latter showed a significantly lower concentration of protein per million cells than Bioreactor MTFs (Figure 2a,d). IL-6 production per mL was lower in the Bioreactor and Monol Reg Vol and significantly higher in Monol Low Vol (Figure 2b). No statistical differences were found when comparing the production of IL-6 per million cells among the three MTFs (Figure 2e). The PGE2 concentration was not significantly different among the MTFs (Figure 2c,f).

### 3.2. The 3D Dynamic Culture-Produced MTFs Partially Inhibited PHA’s Effect on PBMNCs

**Figure 3:**
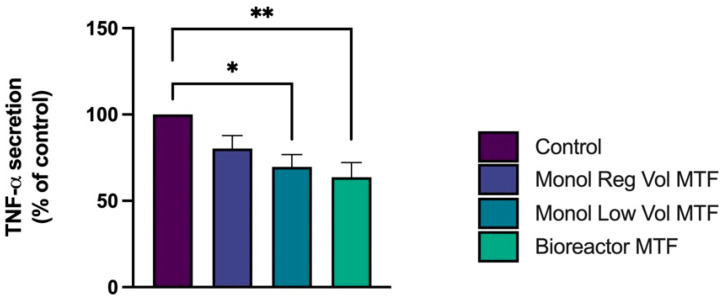
**Inhibition of the inflammation response by MTFs in the PHA agglutination assay on PBMNCs.** PBMNCs were treated for 48 h with PHA. Then the MTFs produced under different culture conditions were introduced. TNF-α was detected in the collected supernatants using ELISA. The levels of TNF-α are reported as a percentage of the control, which was not treated with MTFs. * *p* < 0.05, ** *p* < 0.01. Error bars represent SEM.

For the PHA assay, MTFs from bioreactor and monolayer low volume cultures reduced the release of TNF-α by 36.3% and 30.3%, respectively (Figure 3a). The 3D microcarriers culture in spinner flask MTFs (Bioreactor MTFs) had a greater effect inhibiting inflammation in PBMNCs when compared to the control than Monol Low Vol MTFs, and the Monol Reg Vol MTFs effect did not change the TNF-α secretion in a statistically significant manner.

### 3.3. TNF-α Release Is Decreased by MTFs in LPS-Activated THP-1 Macrophages

**Figure 4:**
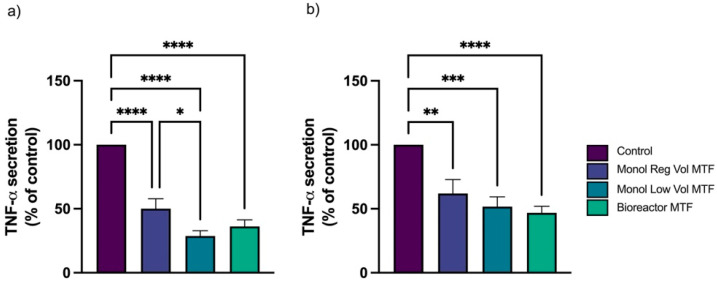
**Inflammation inhibition based on TNF-α detected by ELISA on the LPS-stimulated macrophages.** THP-1-derived macrophages stimulated with LPS were exposed to the different MTFs. TNF-α secreted by the macrophages was measured by ELISA under Treatment 1 (T1) (**a**) and Treatment 2 (T2) (**b**) conditions. The levels of TNF-α are reported as a percentage of the control (not treated with MTFs). Control activated (with LPS) without MTFs is 100%, n = 9. * *p* < 0.05, ** *p* < 0.01, *** *p* < 0.005, and **** *p* < 0.001. Error bars represent SEM.

When Bioreactor MTFs obtained from 3D microcarriers MSCs in spinner flask dynamic culture were added together with LPS (T1), we observed an average inflammation inhibition of 61% based on TNF-α release. However, when the same MTFs were added after LPS (T2), a 50% reduction in inflammation was observed. In T1, the effects of the 3D microcarriers dynamic culture-produced MTFs (Bioreactor MTFs) were similar to those of the monolayer-produced MTFs (Figure 4a). Under T2, the effect of MTFs from the Monol Reg Vol culture (*p* < 0.01) and Monol Low Vol culture (*p* < 0.005) was slightly smaller than that of the Bioreactor MTFs (*p* < 0.001) (Figure 4b). An MTT viability assay showed no significant differences between macrophages treated or untreated with MTFs (Supplementary Figure S5). Although a general reduction in viability was observed in all the wells treated with LPS, the viability remained above 50%.

### 3.4. Inflammatory Cytokines Are Inhibited by MTFs, and the Effect Is Greater with 3D Dynamic Culture

**Figure 5:**
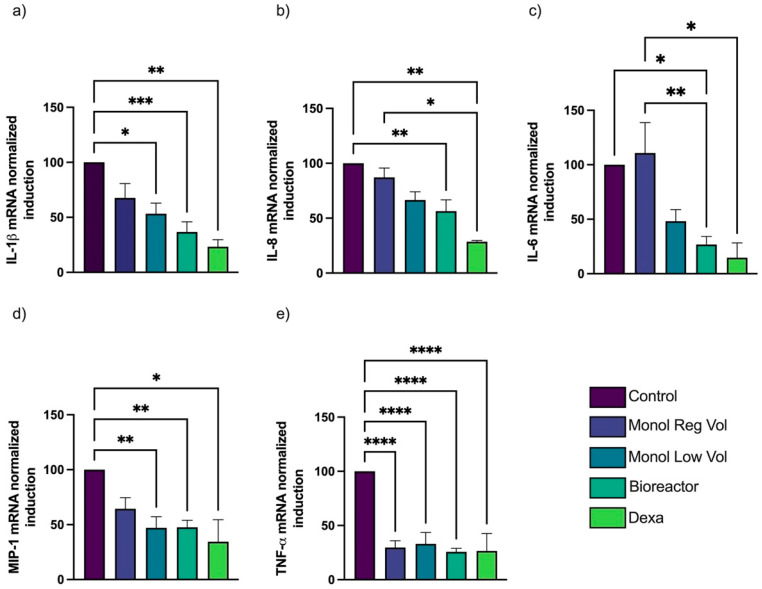
**Comparison of gene expression for T1 experiments; Control values set to represent 100%.** Expression of inflammatory cytokines by THP-1-derived macrophages exposed to LPS and MTF or dexamethasone (positive control) for 5.5 h. The y-axes represent the percentage of folding mRNA expression compared to that obtained by the control without MTF, set as 100%. Bioreactor MTF presented a significant reduction in IL-1β (**a**), IL-8 (**b**), IL-6 (**c**), MIP-1 (**d**), and TNF-α (**e**) compared to the control. MTF from Monol Low Vol also significantly reduced the expression of IL-1β, MIP-1, and TNF-α. MTF produced by Monol Reg Vol only significantly affected the expression of TNF-α. Error bars represent standard error of the mean (SEM); * *p* < 0.05; ** *p* < 0.01; *** *p* < 0.001; **** *p* < 0.0001.

**Figure 6:**
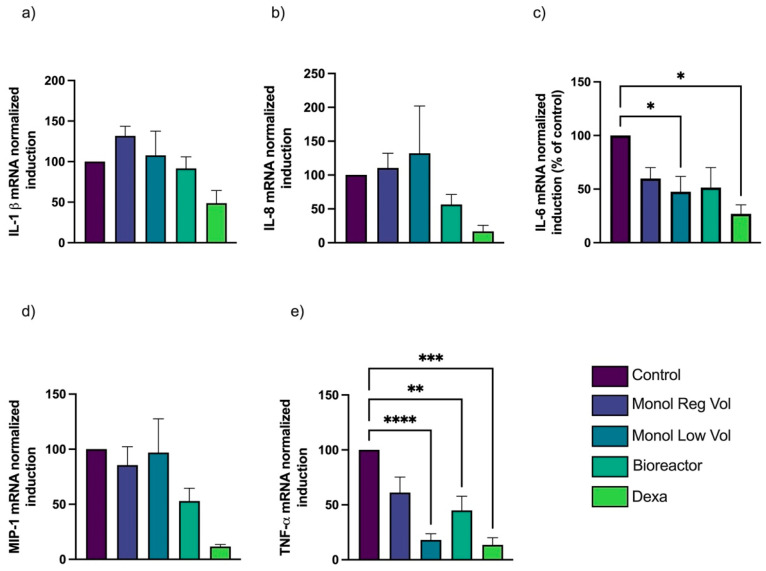
**Comparison of gene expression for T2 experiments; Control values set to represent 100%.** Expression of inflammatory cytokines by THP-1-derived macrophages exposed to LPS for 90 min, followed by the addition of MTFs or dexamethasone (positive control) for 4 h more (together with LPS). The y-axes represent the percentage of folding mRNA expression compared to that obtained by the control without MTFs, set as 100%. Bioreactor MTFs resulted in a significant reduction of only TNF-α compared to the control (**e**). Monol Low Vol significantly inhibited the expression of IL-6 (**c**) and TNF-α (**e**) compared to the control. There were no significant differences in IL-1β (**a**), IL-8 (**b**), or MIP-1 (**d**) expression when compared to the control. Monol Reg Vol-derived MTFs did not present a significant reduction in the expression of any of the cytokines. Error bars represent standard error of the mean (SEM); * *p* < 0.05; ** *p* < 0.01; *** *p* < 0.001, **** *p* < 0.0001.

An RT-PCR was performed for IL-1β, IL-8, TNF-α, MIP-1, and IL-6, using β-Actin as the reference gene. There were no significant differences in cytokine levels among the samples without LPS (−LPS). Moreover, no signs of overexpression of these inflammatory cytokines were found for T1 or T2 in macrophages treated or untreated with MTFs (Supplementary Figures S6 and S7). MTFs induced a clear inhibitory effect on the expression of all the inflammatory cytokines measured (Figure 5 and Figure 6, normalized using relative expression compared to control). Bioreactor MTFs and the Monol Low Vol culture (more concentrated cell/mL) demonstrated a greater inhibitory effect. This effect was more evident in Treatment 1 (T1), where IL-1β, IL-6, MIP-1, and TNF-α were reduced by more than 50% by both Monol Low Vol and Bioreactor MTFs (Figure 5); however, this reduction was only found to be statistically significant for IL-1β, MIP-1, and TNF-α when using the Bioreactor and Monolayer MTFs, while IL-8 and IL-6 were only significantly affected by the Bioreactor MTFs.

Under T2, a reduction in cytokine expression was observed, but the variances were greater, and the MTF effect showed a general decrease. Statistically significant differences were seen only for TNF-α expression, for MTF Monol Low Vol (*p* < 0.0001), and Bioreactor (*p* < 0.01). Also, there was a significant difference in the effect on IL-6, only for Monol Low Vol MTFs (*p* < 0.05) (Figure 6).

A Principal Component Analysis (PCA) for T1 was used to identify molecules whose concentration explained most of the variability in the data. TNF-α, IL-8, and IL-6 were shown to be the molecules most affected by Bioreactor MTFs in the LPS-stimulated macrophages inflammation in vitro model (Supplementary Figure S8).

### 3.5. The 3D Dynamic Culture Produces TNF-α Inhibitory MTFs More Efficiently Than Monolayer

Taking into consideration the macrophage assay using T1 conditions and analyzing the aggregated ELISA and mRNA expression results (Table 2), the MTFs from the Monol Reg Vol cultures presented less TNF-α inhibitory activity than MTFs from the Monol Low Vol or 3D dynamic cultures.

**Figure 7:**
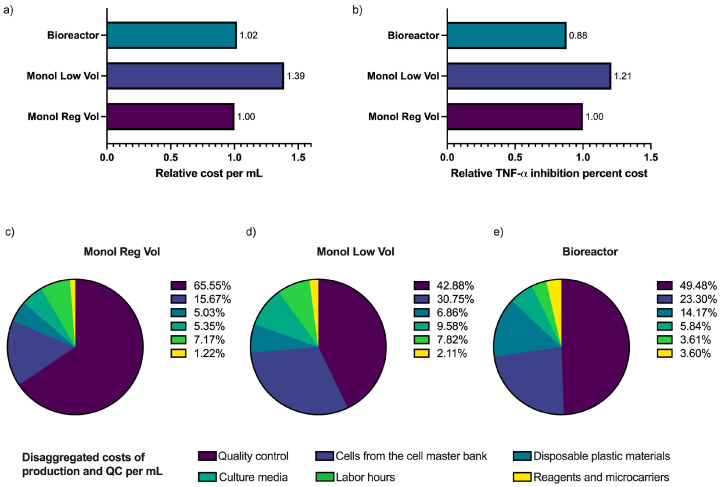
**Comparison of production costs relative to 1 mL of Monol Reg Vol and the cost of one-percent inhibition of TNF-α.** (**a**) Represents the cost of the production and QC relative to Monol Reg Vol per mL and (**b**) relative TNF-α Inhibition percent cost compared to Monol Reg Vol. (**c**–**e**) The pies represent the distribution of the costs by category, as explained in the Materials and Methods section.

The cost per 1 mL of MTFs considered the direct costs of the quantity of cells used from the cell master bank, materials, culture media, reagents and microcarriers, and labor time, plus the costs of quality control. For this study, the cost presented per mL is relative to the cost of 1 mL of Monol Reg Vol MTFs. The cost per mL was very similar between Monol Reg Vol MTFs and Bioreactor MTFs, but the cost of production per inhibition percent was 11.5% and 41.5% higher for Monol Reg Vol and Monol Low Vol, respectively, when compared to the production costs using 3D dynamic culture (Bioreactor) (Figure 7).

Within our experimental setting, the major costs for monolayer production are associated with culture medium consumption (810 mL in monolayer vs. 300 mL in bioreactor) and labor time (12 h in monolayer vs. 4 h in bioreactor). The major costs associated with the bioreactor culture are the disposable bioreactor, metabolite monitoring supplies, and microcarriers. Quality control is the same for all, but since the 3D dynamic culture batch is larger, the cost per mL is diluted (Figure 7).

## 4. Discussion

We have described a reproducible method for obtaining MSCs with a high viability and doubling rate. The average 23-fold increase we measured over a 7-day culture is similar to previous reports [40,41]; however, the method reported here has additional advantages. For example, the cells can be promptly inoculated from the cryo-freezer without a pre-culture step, which is an advantage for scaling up the use of closed systems. Macroporous microcarriers can also reduce the lag phase by protecting the cells and reducing shear stress, without the need for surfactants [42], which can improve yield [43]. We maintained a low-glucose culture, as a high-glucose culture medium may decrease the proliferation capacity and induce premature senescence [44,45]. Similarly, serum deprivation was used for obtaining MTFs to enhance the immunomodulatory capacity of the cells [46]. Using three-dimensional microcarriers allows for a tridimensional arrangement, which eliminates the disadvantage of the low viability of spheroids and the limitations associated with long-term culture [27]. Therefore, our results demonstrate the advantages of three-dimensional cell culture, which more closely mimics the normal in vivo physiologic environment of MSCs; this could improve the cellular therapeutic functionality of culture-expanded MSCs [47]. The reduced storage and handling requirements of a cell-free final product, coupled with the reduced cost of production, would make the therapy dose more affordable from a clinical perspective. Therefore, the main foci of this study were to analyze the potency and costs of cell-free MTF products in 2D and 3D systems. 

The MTF quality control included IL-6, total protein, and PGE2 concentration analyses. It was not expected to see a change in IL-6, but it was included as a safety measure, since the increasing of IL-6 in the conditioned medium could be pro-inflammatory and pro-tumorigenic [48,49]. Bioreactor conditions did not alter IL-6 production, but it was significantly higher in the low-volume monolayer MTFs, likely due to the extra stress cells they were exposed to in terms of nutrient limitation. This suggests that reducing volume is not enough to increase the potency of conditioned medium. PGE2 production was chosen since it has been described as a key molecule of the immune-modulatory effect of MSCs and MSC-CM, [50,51]. The concentration of PGE2 and production per cell were not significantly different among treatments, and 3D microcarrier-based spinner flask dynamic cultured cells produced higher amounts of total protein. Further proteomic analysis would be useful to understand the differences among the different MTFs.

In terms of TNF-α measured in the PHA-activated PBMNCs, Bioreactor MTFs and Monol Low Vol MTFs showed comparable levels of inflammation inhibition (36.3% and 30.3%, respectively). These numbers are comparable to previous studies that reported inhibition of 36.72% using exosomes from one million MSCs in PHA-stimulated cells [35]. These percentages are within the range reported for MSC co-cultures (27–88%) [52,53,54]. This establishes the repeatability of the test and shows that the effect of isolated EVs is similar to that of using the whole MTF. Thus, with our approach, it may not be necessary to invest in further downstream processes to obtain the desired anti-inflammatory effect.

The second model of MTF potency involved macrophages [33,36]. Gene expression results demonstrated a general decrease in inflammatory cytokines when using MTFs in both T1 and T2 (Figure 4, Figure 5 and Figure 6), and MTFs from the low-volume monolayer and bioreactor cultures showed the strongest effect. Dexamethasone was used as a control, as it is a commonly used synthetic glucocorticoid with potent anti-inflammatory properties. However, the long-term use of corticoids is associated with numerous adverse events and increases the risk of infections [55,56,57]. In this study, we observed that the Bioreactor MTFs exhibited statistically similar effectiveness in reducing inflammation compared to the dexamethasone control, which is promising in terms of developing a potential clinical treatment with comparable benefits, but significantly fewer adverse events.

Our MTFs (not pre-conditioned with inflammatory stimuli or hypoxia) exhibited a broad anti-inflammatory effect on activated macrophages. The establishment of functional potency release criteria is critical for assessing the intra-lot variability of biologics [58]. Assays that can measure the potency of MSCs and their secretome (MTF) in inflammatory settings on macrophages have gained particular interest among our group and other researchers [59]. Based on the results of the PCA analysis, we propose quantifying the modulation of the cytokines TNF-α, IL-8, and IL-6 as a means to evaluate the anti-inflammatory potential of drug candidates under T1 conditions. The reduction of these molecules has clinical relevance, such as drugs targeting TNF-α and IL-6 for the treatment of chronic inflammatory conditions. Anti-TNF agents, like adalimumab, etanercept, and certolizumab, inhibit cytokines and promote inflammation resolution [60]. In the management of rheumatoid arthritis, reducing IL-6 in joints has shown benefits [61]. Monoclonal antibodies directed against IL-6, including tocilizumab, sarilumab, and others, have been suggested for rheumatoid arthritis treatment. IL-8 is implicated in various inflammatory conditions, with increased expression of IL-8 and/or its receptors observed in many chronic inflammatory diseases, often correlating with diseases’ activity [62]. IL-8 has been suggested as a target for anti-cancer drugs [63,64] and has been associated with adverse outcomes in COVID-19 patients, along with TNF-α, IL-6, IL-1β, and IL-33 [65]. In the context of cystic fibrosis clinical trials, the IL-8 delta CT post-LPS effect of MSC-CM was assessed as a potency predictor for selecting MSC lots [66]. Therefore, analyzing TNF-α, IL-6, and IL-8 seems reasonable to evaluate anti-inflammatory responses, and further studies could investigate the correlation between the in vitro inhibition of these cytokines and the in vivo anti-inflammatory effects in patients.

The expression of cytokines in macrophages following LPS priming in Treatment 2 (T2) revealed that the effect of MTFs was less immediate or less effective compared to the RNA expression results in T1. Bioreactor MTFs under T2 conditions showed a significant reduction in TNF-α expression only. This suggests that to achieve a clinically relevant product, it may be necessary to use pre-conditioned MSCs or administer multiple doses. In MSC therapy, the presence of inflammatory markers in the patient could stimulate the display of an anti-inflammatory response by MSCs. However, in the case of a cell-free product, suppressing ongoing inflammation may require adjusting the dose according to the patient’s inflammation level, which would require utilizing precision medicine tools or employing pre-conditioning with pro-inflammatory agents to induce MSCs to secrete more anti-inflammatory factors [22]. These aspects could be further investigated in Phase I clinical trials.

Cost analysis is crucial for a product to advance into a drug or a medicinal product. In our study, we found that the cost of manufacturing MTFs in the 3D microcarrier in spinner flask dynamic culture was equivalent to that of the monolayer method. Further scalability and process optimization could potentially reduce production costs even further when utilizing bioreactors, especially considering that their widespread use is still in the early stages [67]. Bioreactors offer several advantages, such as automation, reduced consumption of culture media, decreased labor requirements, and, consequently, lower costs [68], which correlated with our process cost evaluation. Additionally, the closed-system nature of semi-automated and automated bioreactors minimizes the risk of human error and exposure to contaminants, thereby enhancing product quality and reducing infrastructure requirements [68].

Some limitations of this study include the lack of extracellular vesicles (EVs) analysis in the MTFs, which means we did not determine the role of EVs in the differential effects observed among the bioreactor and monolayer cultures. Additionally, important molecules, such as IL-10, IL-1RA, IL-13, TGF-B1 [21], and TSG-6 [69], could have been included in the potency experiments. In future experiments, we plan to use recombinant human gelatin microcarriers to continue developing clinical production methods and explore the effect of priming with pro-inflammatory molecules or hypoxia, as previously discussed [22].

Conditioned medium derived from MSCs is already being used in various clinical trials for skin and bone regeneration, as well as in the treatment of multiple sclerosis via different application routes, including intraarticular, intra-nasal, or intra-muscular delivery [70,71]. Our results are expected to contribute to the establishment of standards for comparing culture systems in future applications of therapeutical products for autoimmune conditions or chronic inflammatory diseases affecting different organs and tissues. One potential application could be the treatment of COVID-19 and long COVID, which involve cytokine storms and persistent inflammation, respectively, and continue to affect millions of individuals worldwide [72]. Mallis et al. reviewed the mechanisms of action and presented evidence of using a COVID-19-positive patient’s serum to stimulate MSCs as a new way of priming and possibly improving the therapeutical products [16,21,73]. Our data can contribute to paving the way for cost-effective manufacturing of clinically relevant MSC-derived products.

## 5. Conclusions

The results of the present study demonstrate a highly reliable method for obtaining MSCs with a high viability and doubling rate in a 3D dynamic system with macroporous gelatin microcarriers in a low-glucose medium. We have successfully shown the simultaneous production of MSCs and anti-inflammatory MTFs, without the need for additional concentration or purification steps. The use of MTFs produced in a 3D dynamic system is more cost-effective per anti-inflammatory percent unit compared to MTFs obtained from a monolayer culture. Furthermore, we have developed a method to quantify the anti-inflammatory effect of MTFs, which can serve as a reference for quality control procedures and contribute to improving the efficacy of the product for clinical use. The development of enhanced MTF products of clinical relevance in a cost-effective manner holds the potential to offer therapeutic solutions with fewer adverse effects compared to the drugs currently available for the treatment of acute and chronic inflammation, as well as immune disorders.

## Figures and Tables

**Table 1:** Doubling rate and viability for the three replicates of three lots cultured in the 3D dynamic system, according to the cell count at the time of harvest. No significant differences were observed between lots, as evidenced by the low coefficients of variance (CVs) percentages.

	Average Viability (%)	Viability CV (%)	Average Doubling Time (h)	Doubling Time CV (%)
**Lot 1**	91.10	3.71	31.49	11.84
**Lot 2**	85.67	4.82	30.58	8.92
**Lot 3**	86.20	0.91	31.21	2.80

**Table 2:** The percentage of inhibition of the inflammation of MTFs from monolayer culture (regular or low volume) or from bioreactor, using the production and mRNA expression of TNF-α, measured by ELISA and RT-PCR, respectively, in the macrophages under T1 conditions.

	Inhibition Percentage *		
	Monol Reg Vol	Monol Low Vol	3D Dynamic Culture
*TNF-α* *from ELISA*	49.96	71.36	63.86
*TNF-α* *from mRNA RT-PCR*	70.30	66.90	75.40
***Sum***	**120.26**	**138.26**	**139.26**

* Inhibition percentage is obtained by subtracting the expression of the inflammatory molecule from 100.

## Data Availability

The data presented in this study are available in the article and supplementary material.

## Supplementary Materials

The following supporting information can be downloaded at: https://www.mdpi.com/article/10.3390/bioengineering10080930/s1, Figure S1: Immuno-phenotype characterization of UC-MSC. Figure S2: UC-MSCs showed differentiation potential. Figure S3: Morphology and attachment evaluation. Figure S4: Viability and confluence eye-inspection. Figure S5: Viability percentage of cells with or without LPS and MTF or dexamethasone; Figure S6: mRNA fold induction of inflammatory cytokines in cells treated with MTF in the presence or absence of LPS (Treatment 1). Figure S7: mRNA fold induction of inflammatory cytokines in cells treated with MTF in the presence or absence of LPS (Treatment 2). Figure S8: Principal Component Analysis (PCA).

## Appendix A. Potency Assays of MTFs from Monolayer and Bioreactor Systems

MTFs from the monolayer and bioreactor systems were subjected to potency tests in three setups: in a system of PBMNC activated with phytohemagglutinin (PHA) (1) and in a system with THP-1-derived macrophages activated with lipopolysaccharides (LPS) added at the same time as the MTFs (Treatment 1) (2) or before the MTFs (Treatment 2) (3).

### Appendix A.1. PHA Inflammation Assay

PBMNCs were isolated by a density gradient with lymphocyte separation medium (Lonza) from a single donor’s peripheral blood (IRB approval number: IRCM-2023-372). A total of 2.4 × 10^6^ viable cells were added to each well in six-well plates, which were then incubated with PHA 5 μg/mL, using 50% of RPMI 1640 supplemented with 10% FBS and 4 mM GlutaMax and 50% of MTFs for 48 h. Supernatants were recovered and saved for TNF-α ELISA tests using an EASIA kit (Novex, Thermo).

### Appendix A.2. THP-1 Macrophages Differentiation and LPS Assay

THP-1 (human monocyte cell line) cells, provided by INDICASAT-AIP, were cultured using the procedure described by Daigneault et al. [74]. In brief, PMA was added to a final concentration of 50 ng/mL in RPMI medium without phenol red, supplemented with 2 mM of *l*-glutamine (Lonza) and 10% FBS (Gibco). Cells were incubated in this medium for 72 h. After that period, most of the cells were attached. Then, the medium was replaced with RPMI with 2 mM of *l*-glutamine (Lonza) and 10% FBS (Gibco), without PMA, and cells were cultured for five more days. Cells were then characterized by flow cytometry using CD11b and CD45 (BD Pharmingen) (double-positive population >80%). An MTT test was performed to eliminate bias due to differences in viability (results in supplementary information).

The potency assay using macrophages was modified from Bartosh et al. [36,75] and similar to the procedure described by Pacienza et al. [33]. MTF was added to the macrophage system either with LPS (Treatment 1, T1) or after LPS (Treatment 2, T2). The T1 assay lasted 5 h and 30 min (5.5 h), with MTFs at 50% and LPS at a final concentration of 100 ng/mL. T2 had a previous incubation of 90 min, with LPS at 100 ng/mL, after which the medium was replaced by 50% MTFs and LPS 100 ng/mL, and then incubated four more hours. In both treatments, the total time of incubation with LPS was 5.5 h. Three controls were used: a positive control for inflammation of LPS 100 ng/mL, the control for anti-inflammatory effects using dexamethasone 10 µg/mL, and the blank using only the matrix (RPMI medium with 2 mM GlutaMax) as a negative control. The supernatant was collected for TNF-α assays using the EASIA kit (Novex, Thermo). The macrophage monolayer was used for the MTT viability assay or lysated using Tri-Reagent (Ambion, Thermo) for subsequent mRNA extraction.

### Appendix A.3. Real-Time PCR Analysis

Total RNA was extracted using TRI Reagent™ Solution (Invitrogen, AM9738). cDNA was generated from 1 μg total RNA in a reaction with the High-Capacity cDNA reverse transcription kit with RNAse inhibitor (Applied Biosystems 4374966). RT-PCR was performed on an ABI 7500 (Applied Biosystems) using SYBR Green, following the manufacturer’s instructions and optimization procedures described by Gonzalez et al. [76]. Amplification conditions were as follows: 95 °C (10 min), 40 cycles of 95 °C (15 s), and 60 °C (60 s). β-Actin expression served as the reference gene for the relative quantification. The following primer sequences were used (from Integrated DNA Technologies, custom-made oligos):

β-actin forward: 5′-ATTGCCGACAGGATGCAGAA-3′
β-actin reverse: 5′-GCTGATCCA CATCTGCTGGAA-3′
IL-6 forward: 5′-TTCAATGAGGAGACTTGCCTG-3′
IL-6 reverse: 5′-ACAACAACAATCTGAGGTGCC-3′
IL-8 forward: 5′-AAGGAACCATCTCACTGTGTGTAAAC-3′
IL-8 reverse: 5′-ATCAGGAAGGCT GCCAAGAG-3′
MIP-1α forward: 5′-TTGTGATTGTTTGCTCTGAGAGTTC-3′
MIP-1α reverse: 5′-CGG TCGTCACCAGACACACT-3′
IL-1β forward: 5′-TACAAGGAGAAGAAAGTAATGACAA-3′
IL-1β reverse: 5′-AGCTTGTTATTGATTTCTATCTTGT-3′
TNF- α forward: 5′-CTCCTCACCCACACCATCAGCCGCA-3′
TNF- α reverse: 5′-ATAGATGGGCTCATACCA GGGCTTG-3′
