# Supplementary material for: Comparison of Cost and Potency of Human Mesenchymal Stromal Cell Conditioned Medium Derived from 2- and 3-Dimensional Cultures
Source: Bioengineering (Basel). 2023 Aug 4;10(8):930. doi: 10.3390/bioengineering10080930 (PMC10451979; doi:10.3390/bioengineering10080930)
Supplement: Supplementary file 1 [file bioengineering-10-00930-s001.zip › Figure S1.pdf]

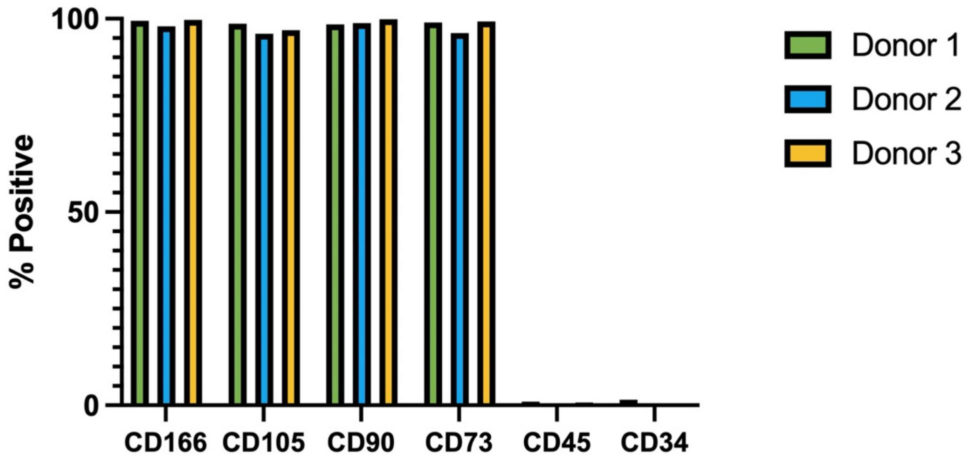

Figure S1. Immuno-phenotype characterization of UC-MSC. MSC's cell surface markers expression identity was demonstrated by positive detection (higher than 95%) of conjugated antibodies for CD90, CD73, CD105, CD166, and negative expression of CD34 and CD45 (less than 2%) on the three lots derived from three different donors used for this work.
