# Supplementary material for: Comparison of Cost and Potency of Human Mesenchymal Stromal Cell Conditioned Medium Derived from 2- and 3-Dimensional Cultures
Source: Bioengineering (Basel). 2023 Aug 4;10(8):930. doi: 10.3390/bioengineering10080930 (PMC10451979; doi:10.3390/bioengineering10080930)
Supplement: Supplementary file 1 [file bioengineering-10-00930-s001.zip › Figure S3.pdf]

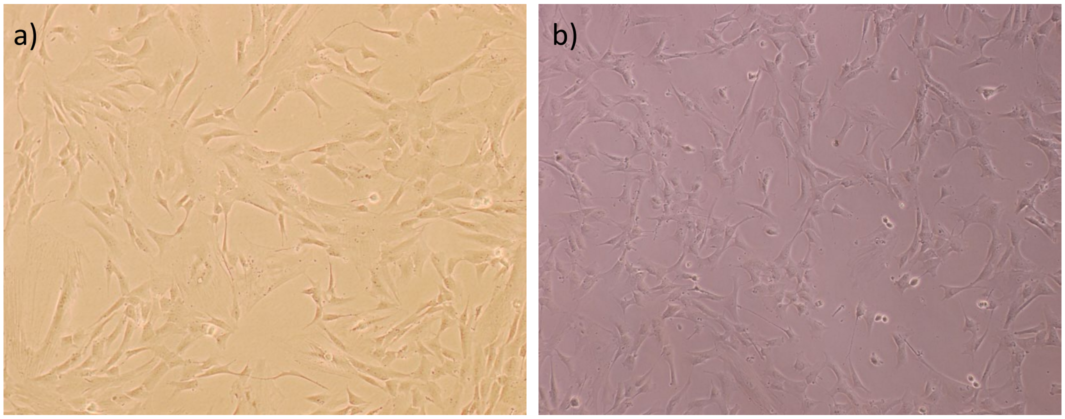

Figure S3. Morphology and attachment evaluation. Frozen MSC's were thawed and plated to evaluate attachment and morphology. At the inverted microscope (10X) cells from monolayer (a), and from bioreactor (b), did not show differences.
