# Supplementary material for: Comparison of Cost and Potency of Human Mesenchymal Stromal Cell Conditioned Medium Derived from 2- and 3-Dimensional Cultures
Source: Bioengineering (Basel). 2023 Aug 4;10(8):930. doi: 10.3390/bioengineering10080930 (PMC10451979; doi:10.3390/bioengineering10080930)
Supplement: Supplementary file 1 [file bioengineering-10-00930-s001.zip › Figure S4.pdf]

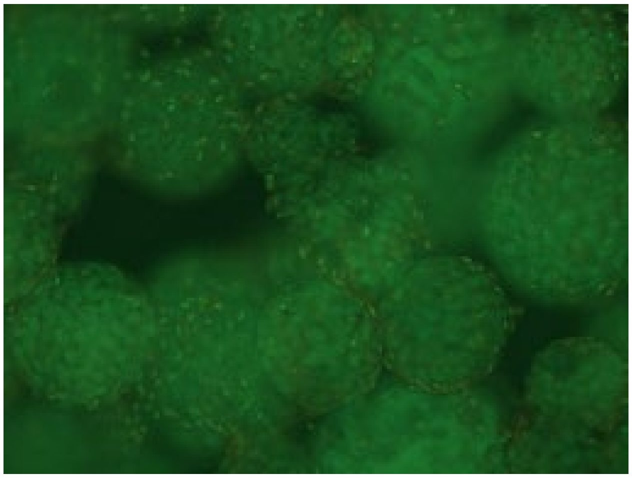

Figure S4. Viability and confluence eye-inspection. A small sample of the bioreactor was collected and stained using Acridine orange, to evaluate growth and absence of apoptotic cells. This picture was captured on day 6 of culture under (4X) inverted microscope.
