# Supplementary material for: Comparison of Cost and Potency of Human Mesenchymal Stromal Cell Conditioned Medium Derived from 2- and 3-Dimensional Cultures
Source: Bioengineering (Basel). 2023 Aug 4;10(8):930. doi: 10.3390/bioengineering10080930 (PMC10451979; doi:10.3390/bioengineering10080930)
Supplement: Supplementary file 1 [file bioengineering-10-00930-s001.zip › Revised Figure S2.pdf]

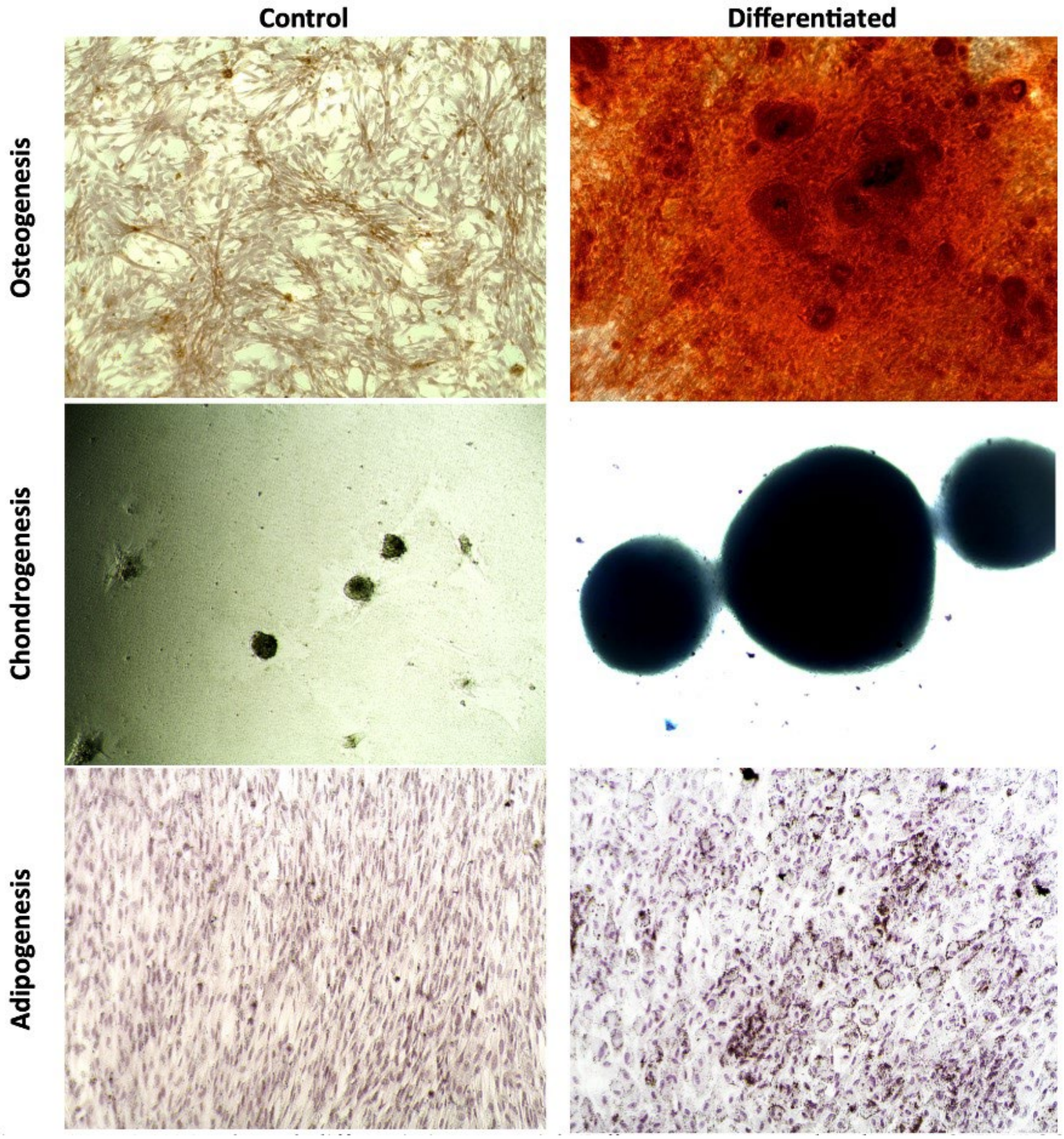

Figure S2. UC-MSCs showed differentiation potential. Differentiation was induced using STEMPRO® osteogenic, chondrogenic and adipogenic medium respectively. Cells were stained using alizarin red, alcian blue and oil red counterstained with hematoxylin, respectively. Pictures were captured using 10x objective in an inverted microscope.
