# Supplementary material for: Comparison of Cost and Potency of Human Mesenchymal Stromal Cell Conditioned Medium Derived from 2- and 3-Dimensional Cultures
Source: Bioengineering (Basel). 2023 Aug 4;10(8):930. doi: 10.3390/bioengineering10080930 (PMC10451979; doi:10.3390/bioengineering10080930)
Supplement: Supplementary file 1 [file bioengineering-10-00930-s001.zip › Revised Figure S5.pdf]

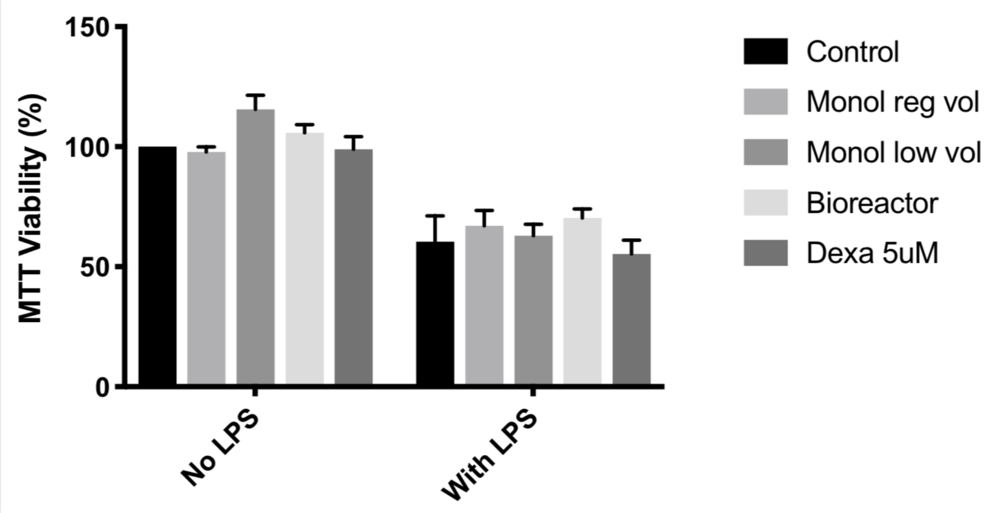

Figure S5. Viability percentage of cells with or without LPS and MTF or dexamethasone. A higher viability percentage was observed for the THP-1 derived macrophages without LPS. The viability of macrophages was reduced following 5.5 hours of incubation with LPS, but remained above 50% according to an MTT assay. There were no significant differences in viability among different MTFs or when compared with dexamethasone and the control.
