# Supplementary material for: Comparison of Cost and Potency of Human Mesenchymal Stromal Cell Conditioned Medium Derived from 2- and 3-Dimensional Cultures
Source: Bioengineering (Basel). 2023 Aug 4;10(8):930. doi: 10.3390/bioengineering10080930 (PMC10451979; doi:10.3390/bioengineering10080930)
Supplement: Supplementary file 1 [file bioengineering-10-00930-s001.zip › Revised Figure S6.pdf]

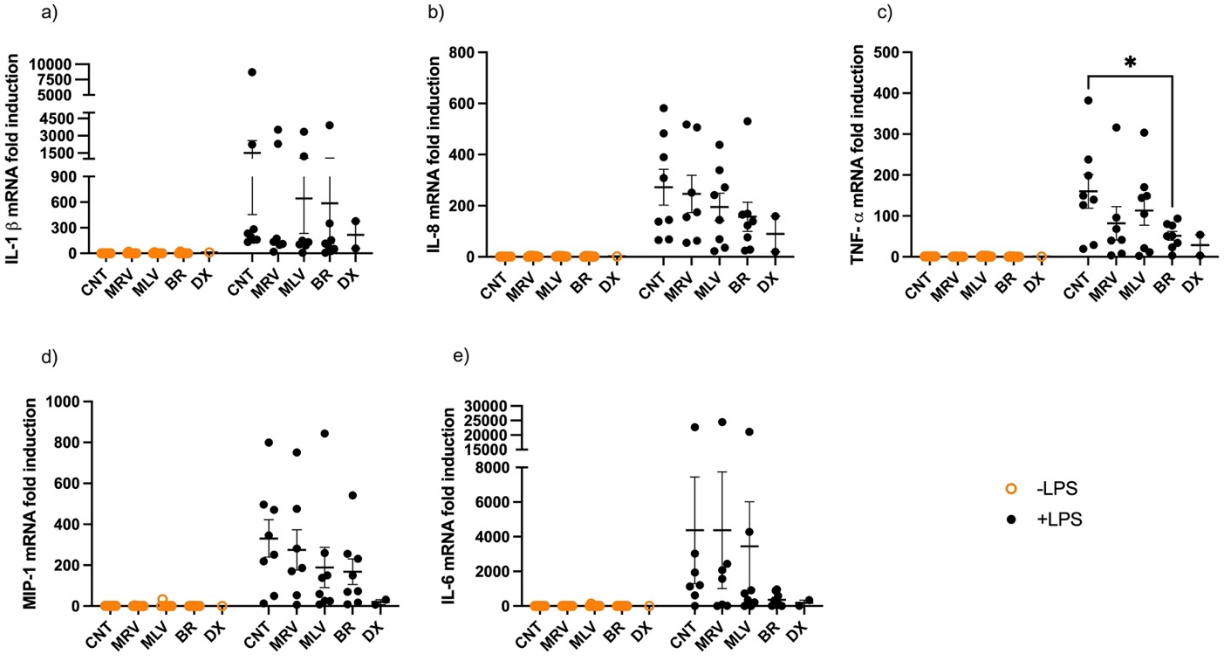

Figure S6. mRNA fold induction of inflammatory cytokines in cells treated with MTF in the presence or absence of LPS (Treatment 1). Semi-quantitative gene expression was determined using the Ct value, which was calculating through delta delta Ct analysis ($e^{-\Delta\Delta Ct}$) obtained from the RT-PCR results (using the procedure described in the methods). There was no evidence of inflammation using only MTF or dexamethasone. With LPS, all the samples expressed high levels of IL-1β (a), IL-8 (b), TNF-α (c), MIP-1 (d) and IL-6 (e). Treatment with Bioreactor MTF resulted in a significant reduction in TNF-α only; significant differences were not observed for the other cytokines tested or the monolayer MTFs tested. Bars represent standard error of the mean (SEM); * $p<0.05$. Abbreviations: CNT: Control; MRV: Monolayer Regular Volume; MLV: Monolayer Low Volume; BR: bioreactor; DX: Dexamethasone (positive control).
