# Supplementary material for: Comparison of Cost and Potency of Human Mesenchymal Stromal Cell Conditioned Medium Derived from 2- and 3-Dimensional Cultures
Source: Bioengineering (Basel). 2023 Aug 4;10(8):930. doi: 10.3390/bioengineering10080930 (PMC10451979; doi:10.3390/bioengineering10080930)
Supplement: Supplementary file 1 [file bioengineering-10-00930-s001.zip › Revised Figure S8.pdf]

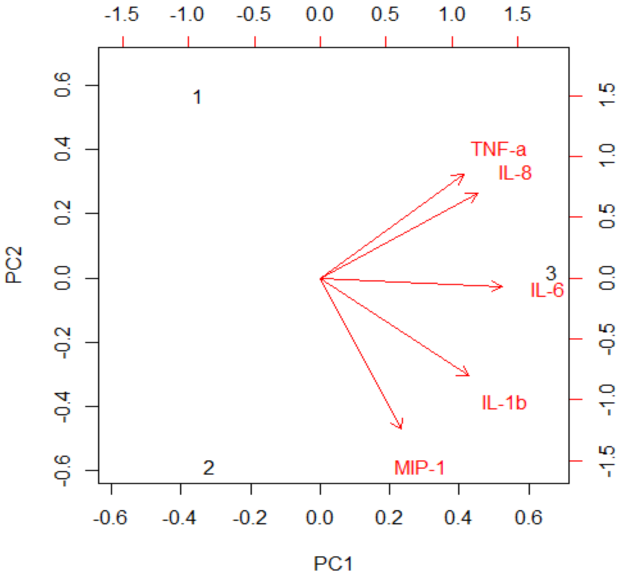

Figure S8. Principal Component Analysis (PCA). Correlation analysis and significance of cytokine concentration-based Treatment 1 lots' replicates. The vectors for IL-6, IL-8 and TNF-α are the most relevant for the anti-inflammatory effects of the bioreactor MTF. There are two main positive vectors in both dimensions of the PCA plot: one for TNF-α and the other for IL-8. The third vector, IL-6, was chosen based on the PCA analysis, but also based on its clinical relevance, because IL-6 is a well-known inflammatory cytokine. IL-1 β and MIP-1 are in the negative part of the plot, which indicates the bioreactor MTF does not affect these cytokines as strongly as the other molecules.
